# Supplementary material for: Multi‐dataset identification of innovative feature genes and molecular mechanisms in keratoconus
Source: J Cell Mol Med. 2024 Sep 19;28(18):e70079. doi: 10.1111/jcmm.70079 (PMC11412914; doi:10.1111/jcmm.70079)
Supplement: Supplementary file 2 — Material S2. [file JCMM-28-e70079-s002.zip › 7.GSEA analysis/4.MYRF/KEGG_CYTOKINE_CYTOKINE_RECEPTOR_INTERACTION.html]

Details for gene set KEGG\_CYTOKINE\_CYTOKINE\_RECEPTOR\_INTERACTION[GSEA]

|  || Dataset | MYRF.group.cls#h\_versus\_l |
| Phenotype | group.cls#h\_versus\_l |
| Upregulated in class | l |
| GeneSet | KEGG\_CYTOKINE\_CYTOKINE\_RECEPTOR\_INTERACTION |
| Enrichment Score (ES) | -0.4971161 |
| Normalized Enrichment Score (NES) | -1.500211 |
| Nominal p-value | 0.009920635 |
| FDR q-value | 0.21524702 |
| FWER p-Value | 0.763 |
Table: GSEA Results Summary

  

Fig 1: Enrichment plot: KEGG\_CYTOKINE\_CYTOKINE\_RECEPTOR\_INTERACTION      
 Profile of the Running ES Score & Positions of GeneSet Members on the Rank Ordered List

  

| SYMBOL | TITLE | RANK IN GENE LIST | RANK METRIC SCORE | RUNNING ES | CORE ENRICHMENT || 1 | LEP | na | 316 | 0.646 | 0.0018 | No |
| 2 | IFNA14 | na | 451 | 0.586 | 0.0079 | No |
| 3 | PF4 | na | 812 | 0.495 | 0.0059 | No |
| 4 | IL21 | na | 1364 | 0.413 | -0.0031 | No |
| 5 | PDGFRA | na | 1428 | 0.407 | 0.0020 | No |
| 6 | IL12A | na | 1851 | 0.369 | -0.0039 | No |
| 7 | IFNL3 | na | 2006 | 0.356 | -0.0023 | No |
| 8 | IFNA6 | na | 2154 | 0.344 | -0.0007 | No |
| 9 | IL4R | na | 2242 | 0.338 | 0.0025 | No |
| 10 | IL26 | na | 2422 | 0.327 | 0.0029 | No |
| 11 | IL17B | na | 2825 | 0.304 | -0.0036 | No |
| 12 | AMHR2 | na | 2900 | 0.300 | -0.0006 | No |
| 13 | IL3RA | na | 3034 | 0.293 | 0.0005 | No |
| 14 | IFNA4 | na | 3565 | 0.269 | -0.0103 | No |
| 15 | IL9 | na | 3726 | 0.263 | -0.0105 | No |
| 16 | CXCR2 | na | 3923 | 0.257 | -0.0118 | No |
| 17 | CXCL13 | na | 4048 | 0.252 | -0.0111 | No |
| 18 | IL20RA | na | 4078 | 0.251 | -0.0077 | No |
| 19 | GH1 | na | 4512 | 0.237 | -0.0162 | No |
| 20 | PPBP | na | 4647 | 0.233 | -0.0161 | No |
| 21 | IL5RA | na | 4719 | 0.231 | -0.0142 | No |
| 22 | TNFRSF19 | na | 4799 | 0.228 | -0.0126 | No |
| 23 | TPO | na | 4921 | 0.225 | -0.0123 | No |
| 24 | CXCL6 | na | 4997 | 0.222 | -0.0107 | No |
| 25 | IL3 | na | 5237 | 0.215 | -0.0140 | No |
| 26 | TNFRSF13C | na | 5257 | 0.215 | -0.0109 | No |
| 27 | FLT3 | na | 5327 | 0.212 | -0.0093 | No |
| 28 | CCL1 | na | 5485 | 0.208 | -0.0103 | No |
| 29 | ACVR2B | na | 5540 | 0.206 | -0.0083 | No |
| 30 | IL7R | na | 5912 | 0.195 | -0.0158 | No |
| 31 | TNFSF8 | na | 6045 | 0.191 | -0.0164 | No |
| 32 | TNFSF13B | na | 6312 | 0.183 | -0.0210 | No |
| 33 | CCR1 | na | 6335 | 0.182 | -0.0185 | No |
| 34 | IFNA16 | na | 6382 | 0.181 | -0.0168 | No |
| 35 | IL2RA | na | 6586 | 0.176 | -0.0196 | No |
| 36 | CCL17 | na | 6752 | 0.171 | -0.0215 | No |
| 37 | CCR6 | na | 7102 | 0.161 | -0.0289 | No |
| 38 | IL13 | na | 7183 | 0.159 | -0.0285 | No |
| 39 | NGFR | na | 7411 | 0.153 | -0.0325 | No |
| 40 | IL18R1 | na | 7436 | 0.152 | -0.0306 | No |
| 41 | BMPR1B | na | 7766 | 0.144 | -0.0377 | No |
| 42 | CCL15 | na | 8571 | 0.125 | -0.0589 | No |
| 43 | IFNE | na | 8811 | 0.120 | -0.0638 | No |
| 44 | FLT4 | na | 9822 | 0.099 | -0.0914 | No |
| 45 | IL23R | na | 9977 | 0.096 | -0.0942 | No |
| 46 | BMPR2 | na | 10193 | 0.093 | -0.0989 | No |
| 47 | IFNL2 | na | 10226 | 0.092 | -0.0982 | No |
| 48 | TNFRSF13B | na | 10434 | 0.088 | -0.1027 | No |
| 49 | XCL1 | na | 10618 | 0.084 | -0.1066 | No |
| 50 | ACVR1B | na | 10708 | 0.083 | -0.1078 | No |
| 51 | PF4V1 | na | 11198 | 0.075 | -0.1207 | No |
| 52 | TNFRSF25 | na | 11215 | 0.075 | -0.1199 | No |
| 53 | CCL23 | na | 11318 | 0.073 | -0.1216 | No |
| 54 | CCR5 | na | 11326 | 0.073 | -0.1206 | No |
| 55 | PLEKHO2 | na | 11808 | 0.066 | -0.1334 | No |
| 56 | IL2RB | na | 12056 | 0.063 | -0.1395 | No |
| 57 | IL17RB | na | 12213 | 0.060 | -0.1430 | No |
| 58 | CCR10 | na | 12225 | 0.060 | -0.1423 | No |
| 59 | CCR4 | na | 12407 | 0.058 | -0.1465 | No |
| 60 | ACVRL1 | na | 12475 | 0.057 | -0.1475 | No |
| 61 | GH2 | na | 12629 | 0.055 | -0.1510 | No |
| 62 | IL21R | na | 12926 | 0.051 | -0.1587 | No |
| 63 | CXCR5 | na | 12998 | 0.050 | -0.1599 | No |
| 64 | INHBC | na | 13034 | 0.050 | -0.1601 | No |
| 65 | CCL26 | na | 13127 | 0.049 | -0.1620 | No |
| 66 | TNFSF4 | na | 13222 | 0.047 | -0.1639 | No |
| 67 | CD70 | na | 13453 | 0.044 | -0.1698 | No |
| 68 | PDGFA | na | 13993 | 0.038 | -0.1848 | No |
| 69 | TNFSF14 | na | 14253 | 0.035 | -0.1917 | No |
| 70 | TNFSF15 | na | 14626 | 0.031 | -0.2020 | No |
| 71 | IFNA2 | na | 14988 | 0.028 | -0.2120 | No |
| 72 | CRLF2 | na | 15203 | 0.026 | -0.2177 | No |
| 73 | IL22 | na | 15308 | 0.025 | -0.2203 | No |
| 74 | IL17RA | na | 15376 | 0.024 | -0.2218 | No |
| 75 | IL20RB | na | 15510 | 0.023 | -0.2253 | No |
| 76 | IL13RA1 | na | 15746 | 0.020 | -0.2318 | No |
| 77 | CCL11 | na | 15898 | 0.019 | -0.2359 | No |
| 78 | TNFRSF14 | na | 15904 | 0.019 | -0.2357 | No |
| 79 | FASLG | na | 16299 | 0.015 | -0.2469 | No |
| 80 | PRL | na | 16445 | 0.013 | -0.2508 | No |
| 81 | CXCL5 | na | 16593 | 0.012 | -0.2549 | No |
| 82 | FLT1 | na | 16677 | 0.011 | -0.2571 | No |
| 83 | CNTFR | na | 16919 | 0.009 | -0.2639 | No |
| 84 | TSLP | na | 17021 | 0.009 | -0.2667 | No |
| 85 | EGFR | na | 17031 | 0.008 | -0.2668 | No |
| 86 | IFNB1 | na | 17252 | 0.006 | -0.2731 | No |
| 87 | XCR1 | na | 17596 | 0.003 | -0.2830 | No |
| 88 | CCL24 | na | 18225 | -0.003 | -0.3012 | No |
| 89 | CCL18 | na | 18351 | -0.004 | -0.3047 | No |
| 90 | IL19 | na | 18360 | -0.004 | -0.3049 | No |
| 91 | CSF3R | na | 18485 | -0.005 | -0.3084 | No |
| 92 | EGF | na | 19142 | -0.012 | -0.3272 | No |
| 93 | IFNAR1 | na | 19408 | -0.015 | -0.3347 | No |
| 94 | TNFRSF10C | na | 20007 | -0.022 | -0.3517 | No |
| 95 | VEGFB | na | 20319 | -0.026 | -0.3602 | No |
| 96 | CXCL14 | na | 20555 | -0.028 | -0.3666 | No |
| 97 | BMPR1A | na | 21219 | -0.036 | -0.3852 | No |
| 98 | TNFRSF1B | na | 21274 | -0.037 | -0.3861 | No |
| 99 | TNFRSF17 | na | 21464 | -0.039 | -0.3910 | No |
| 100 | IL15RA | na | 21497 | -0.040 | -0.3912 | No |
| 101 | KIT | na | 21671 | -0.042 | -0.3955 | No |
| 102 | ACVR2A | na | 21886 | -0.044 | -0.4010 | No |
| 103 | FLT3LG | na | 21921 | -0.045 | -0.4012 | No |
| 104 | CTF1 | na | 22055 | -0.046 | -0.4043 | No |
| 105 | TNFSF9 | na | 22109 | -0.047 | -0.4050 | No |
| 106 | IL18 | na | 22163 | -0.048 | -0.4057 | No |
| 107 | TGFB2 | na | 22177 | -0.048 | -0.4053 | No |
| 108 | BMP7 | na | 22323 | -0.050 | -0.4086 | No |
| 109 | CCL8 | na | 22516 | -0.052 | -0.4133 | No |
| 110 | TNFSF11 | na | 22541 | -0.053 | -0.4131 | No |
| 111 | CCL16 | na | 22560 | -0.053 | -0.4127 | No |
| 112 | TNFSF10 | na | 22572 | -0.053 | -0.4122 | No |
| 113 | IL12B | na | 22624 | -0.054 | -0.4127 | No |
| 114 | CCL27 | na | 22822 | -0.057 | -0.4175 | No |
| 115 | CXCL12 | na | 22943 | -0.059 | -0.4200 | No |
| 116 | EPO | na | 22947 | -0.059 | -0.4190 | No |
| 117 | LTBR | na | 23190 | -0.063 | -0.4250 | No |
| 118 | TNFRSF1A | na | 23281 | -0.064 | -0.4265 | No |
| 119 | LTA | na | 23433 | -0.066 | -0.4298 | No |
| 120 | CCL19 | na | 23536 | -0.068 | -0.4316 | No |
| 121 | KITLG | na | 23576 | -0.069 | -0.4315 | No |
| 122 | CSF1 | na | 23614 | -0.069 | -0.4314 | No |
| 123 | CCR8 | na | 24232 | -0.080 | -0.4480 | No |
| 124 | CXCL16 | na | 24251 | -0.080 | -0.4471 | No |
| 125 | CCR3 | na | 24386 | -0.082 | -0.4496 | No |
| 126 | EDA2R | na | 24800 | -0.090 | -0.4601 | No |
| 127 | TNFSF13 | na | 24888 | -0.091 | -0.4611 | No |
| 128 | TNFRSF4 | na | 24926 | -0.092 | -0.4606 | No |
| 129 | TGFBR2 | na | 24993 | -0.093 | -0.4609 | No |
| 130 | IFNGR1 | na | 25005 | -0.093 | -0.4596 | No |
| 131 | HGF | na | 25029 | -0.093 | -0.4587 | No |
| 132 | TNFRSF10A | na | 25362 | -0.100 | -0.4666 | No |
| 133 | TGFBR1 | na | 25528 | -0.103 | -0.4697 | No |
| 134 | IL22RA1 | na | 25616 | -0.105 | -0.4704 | No |
| 135 | TNFRSF10B | na | 25657 | -0.105 | -0.4698 | No |
| 136 | IL12RB2 | na | 25777 | -0.108 | -0.4714 | No |
| 137 | IL5 | na | 26211 | -0.117 | -0.4819 | No |
| 138 | IL24 | na | 26316 | -0.119 | -0.4829 | No |
| 139 | PDGFB | na | 26504 | -0.123 | -0.4863 | No |
| 140 | TNFSF18 | na | 26839 | -0.131 | -0.4937 | No |
| 141 | CCL28 | na | 26883 | -0.132 | -0.4927 | No |
| 142 | IL10RB | na | 27036 | -0.136 | -0.4948 | Yes |
| 143 | IL6ST | na | 27063 | -0.137 | -0.4932 | Yes |
| 144 | CCL14 | na | 27081 | -0.137 | -0.4914 | Yes |
| 145 | CCR2 | na | 27116 | -0.138 | -0.4900 | Yes |
| 146 | CCL4L2 | na | 27215 | -0.140 | -0.4905 | Yes |
| 147 | PDGFRB | na | 27235 | -0.141 | -0.4886 | Yes |
| 148 | IL6R | na | 27330 | -0.143 | -0.4889 | Yes |
| 149 | LEPR | na | 27355 | -0.144 | -0.4872 | Yes |
| 150 | IFNAR2 | na | 27532 | -0.149 | -0.4898 | Yes |
| 151 | TNFSF12 | na | 27667 | -0.152 | -0.4911 | Yes |
| 152 | IL1R2 | na | 27718 | -0.153 | -0.4899 | Yes |
| 153 | CXCR3 | na | 27781 | -0.155 | -0.4891 | Yes |
| 154 | CCL13 | na | 27880 | -0.157 | -0.4892 | Yes |
| 155 | FAS | na | 28092 | -0.163 | -0.4926 | Yes |
| 156 | GHR | na | 28198 | -0.166 | -0.4928 | Yes |
| 157 | CCR9 | na | 28216 | -0.167 | -0.4905 | Yes |
| 158 | IL11RA | na | 28289 | -0.170 | -0.4897 | Yes |
| 159 | CXCL11 | na | 28291 | -0.170 | -0.4868 | Yes |
| 160 | RELT | na | 28325 | -0.170 | -0.4849 | Yes |
| 161 | IFNLR1 | na | 28478 | -0.174 | -0.4863 | Yes |
| 162 | OSM | na | 28511 | -0.175 | -0.4842 | Yes |
| 163 | IL12RB1 | na | 28594 | -0.178 | -0.4836 | Yes |
| 164 | IFNA7 | na | 28684 | -0.181 | -0.4831 | Yes |
| 165 | TNFRSF12A | na | 28694 | -0.181 | -0.4803 | Yes |
| 166 | MET | na | 28755 | -0.183 | -0.4789 | Yes |
| 167 | CD27 | na | 28772 | -0.184 | -0.4762 | Yes |
| 168 | CNTF | na | 28799 | -0.185 | -0.4738 | Yes |
| 169 | CCL2 | na | 28806 | -0.185 | -0.4709 | Yes |
| 170 | IL10 | na | 28824 | -0.186 | -0.4682 | Yes |
| 171 | CSF1R | na | 28825 | -0.186 | -0.4650 | Yes |
| 172 | IL1R1 | na | 28913 | -0.188 | -0.4643 | Yes |
| 173 | ACVR1 | na | 29069 | -0.193 | -0.4656 | Yes |
| 174 | IL20 | na | 29340 | -0.201 | -0.4700 | Yes |
| 175 | TNFRSF11A | na | 29426 | -0.203 | -0.4690 | Yes |
| 176 | EDAR | na | 29451 | -0.204 | -0.4662 | Yes |
| 177 | IFNA8 | na | 29598 | -0.210 | -0.4669 | Yes |
| 178 | IL2RG | na | 29736 | -0.215 | -0.4672 | Yes |
| 179 | IFNGR2 | na | 29802 | -0.217 | -0.4654 | Yes |
| 180 | VEGFA | na | 29965 | -0.222 | -0.4663 | Yes |
| 181 | AMH | na | 29982 | -0.223 | -0.4630 | Yes |
| 182 | TNFRSF11B | na | 30382 | -0.238 | -0.4705 | Yes |
| 183 | CCL7 | na | 30606 | -0.246 | -0.4728 | Yes |
| 184 | CD40LG | na | 30708 | -0.251 | -0.4714 | Yes |
| 185 | CCL21 | na | 30783 | -0.254 | -0.4693 | Yes |
| 186 | IFNL1 | na | 30883 | -0.257 | -0.4678 | Yes |
| 187 | IL9R | na | 30922 | -0.259 | -0.4644 | Yes |
| 188 | IFNW1 | na | 31073 | -0.268 | -0.4642 | Yes |
| 189 | CCL5 | na | 31086 | -0.268 | -0.4600 | Yes |
| 190 | IL23A | na | 31271 | -0.278 | -0.4606 | Yes |
| 191 | IL1RAP | na | 31283 | -0.279 | -0.4562 | Yes |
| 192 | EDA | na | 31337 | -0.282 | -0.4529 | Yes |
| 193 | CSF3 | na | 31398 | -0.285 | -0.4498 | Yes |
| 194 | EPOR | na | 31475 | -0.290 | -0.4471 | Yes |
| 195 | TNFRSF10D | na | 31642 | -0.298 | -0.4468 | Yes |
| 196 | LTB | na | 31759 | -0.304 | -0.4450 | Yes |
| 197 | TNFRSF8 | na | 31917 | -0.313 | -0.4443 | Yes |
| 198 | TNFRSF9 | na | 32074 | -0.321 | -0.4433 | Yes |
| 199 | CCL22 | na | 32141 | -0.326 | -0.4397 | Yes |
| 200 | IFNA10 | na | 32142 | -0.326 | -0.4341 | Yes |
| 201 | GDF5 | na | 32150 | -0.327 | -0.4288 | Yes |
| 202 | CLCF1 | na | 32202 | -0.329 | -0.4247 | Yes |
| 203 | CXCR6 | na | 32224 | -0.331 | -0.4196 | Yes |
| 204 | INHBB | na | 32328 | -0.338 | -0.4169 | Yes |
| 205 | CSF2RA | na | 32334 | -0.339 | -0.4112 | Yes |
| 206 | TNFRSF6B | na | 32383 | -0.342 | -0.4068 | Yes |
| 207 | IL22RA2 | na | 32410 | -0.344 | -0.4017 | Yes |
| 208 | CD40 | na | 32572 | -0.356 | -0.4003 | Yes |
| 209 | CCR7 | na | 32711 | -0.366 | -0.3981 | Yes |
| 210 | IL18RAP | na | 32770 | -0.370 | -0.3935 | Yes |
| 211 | TNFRSF18 | na | 32782 | -0.371 | -0.3875 | Yes |
| 212 | TGFB1 | na | 32868 | -0.377 | -0.3836 | Yes |
| 213 | CSF2RB | na | 32878 | -0.378 | -0.3774 | Yes |
| 214 | IL1A | na | 32896 | -0.380 | -0.3714 | Yes |
| 215 | IL7 | na | 32965 | -0.385 | -0.3668 | Yes |
| 216 | TNFRSF21 | na | 33002 | -0.388 | -0.3613 | Yes |
| 217 | TGFB3 | na | 33024 | -0.389 | -0.3553 | Yes |
| 218 | IL4 | na | 33045 | -0.391 | -0.3492 | Yes |
| 219 | IL15 | na | 33083 | -0.394 | -0.3436 | Yes |
| 220 | CX3CR1 | na | 33108 | -0.398 | -0.3375 | Yes |
| 221 | CCL3L3 | na | 33123 | -0.399 | -0.3311 | Yes |
| 222 | IFNG | na | 33150 | -0.402 | -0.3250 | Yes |
| 223 | VEGFC | na | 33298 | -0.419 | -0.3221 | Yes |
| 224 | PRLR | na | 33304 | -0.419 | -0.3152 | Yes |
| 225 | KDR | na | 33306 | -0.420 | -0.3080 | Yes |
| 226 | IL6 | na | 33353 | -0.425 | -0.3021 | Yes |
| 227 | IL25 | na | 33355 | -0.425 | -0.2949 | Yes |
| 228 | IL2 | na | 33402 | -0.431 | -0.2889 | Yes |
| 229 | MPL | na | 33691 | -0.468 | -0.2893 | Yes |
| 230 | IL17A | na | 33830 | -0.492 | -0.2850 | Yes |
| 231 | CX3CL1 | na | 33908 | -0.505 | -0.2786 | Yes |
| 232 | LIFR | na | 33932 | -0.510 | -0.2706 | Yes |
| 233 | IL11 | na | 33987 | -0.519 | -0.2633 | Yes |
| 234 | CXCL2 | na | 34010 | -0.524 | -0.2550 | Yes |
| 235 | IFNA21 | na | 34032 | -0.529 | -0.2466 | Yes |
| 236 | IL1B | na | 34248 | -0.586 | -0.2429 | Yes |
| 237 | IFNK | na | 34277 | -0.595 | -0.2336 | Yes |
| 238 | IL10RA | na | 34290 | -0.599 | -0.2238 | Yes |
| 239 | TNF | na | 34328 | -0.609 | -0.2145 | Yes |
| 240 | LIF | na | 34344 | -0.612 | -0.2045 | Yes |
| 241 | INHBE | na | 34413 | -0.640 | -0.1956 | Yes |
| 242 | CSF2 | na | 34421 | -0.642 | -0.1849 | Yes |
| 243 | OSMR | na | 34423 | -0.642 | -0.1740 | Yes |
| 244 | CCL3 | na | 34424 | -0.643 | -0.1630 | Yes |
| 245 | CXCL1 | na | 34529 | -0.693 | -0.1542 | Yes |
| 246 | CXCR4 | na | 34584 | -0.727 | -0.1434 | Yes |
| 247 | PDGFC | na | 34611 | -0.758 | -0.1313 | Yes |
| 248 | CXCR1 | na | 34635 | -0.792 | -0.1185 | Yes |
| 249 | CCL20 | na | 34639 | -0.799 | -0.1050 | Yes |
| 250 | BMP2 | na | 34656 | -0.831 | -0.0913 | Yes |
| 251 | CXCL8 | na | 34657 | -0.838 | -0.0770 | Yes |
| 252 | CCL25 | na | 34662 | -0.842 | -0.0628 | Yes |
| 253 | INHBA | na | 34674 | -0.871 | -0.0483 | Yes |
| 254 | CXCL9 | na | 34681 | -0.884 | -0.0334 | Yes |
| 255 | CXCL3 | na | 34682 | -0.891 | -0.0183 | Yes |
| 256 | CXCL10 | na | 34719 | -1.151 | 0.0003 | Yes |
Table: GSEA details [plain text format]

  

Fig 2: KEGG\_CYTOKINE\_CYTOKINE\_RECEPTOR\_INTERACTION      
 Blue-Pink O' Gram in the Space of the Analyzed GeneSet

  

Fig 3: KEGG\_CYTOKINE\_CYTOKINE\_RECEPTOR\_INTERACTION: Random ES distribution      
 Gene set null distribution of ES for **KEGG\_CYTOKINE\_CYTOKINE\_RECEPTOR\_INTERACTION**
